# An Analysis of the Effects of Noisy Electrocardiogram Signal on Heartbeat Detection Performance

**DOI:** 10.3390/bioengineering7020053

**Published:** 2020-06-06

**Authors:** Ziti Fariha Mohd Apandi, Ryojun Ikeura, Soichiro Hayakawa, Shigeyoshi Tsutsumi

**Affiliations:** 1 Graduate School of Engineering, Mie University, Mie 514-8507, Japan; 2 Department of Mechanical Engineering, Graduate School of Engineering, Mie University, Mie 514-8507, Japan; ikeura@ss.mach.mie-u.ac.jp (R.I.); hayakawa@ss.mach.mie-u.ac.jp (S.H.); tsutsumi@ss.mach.mie-u.ac.jp (S.T.)

**Keywords:** heartbeat detection, noisy signal, ambulatory ECG signal, ECG analysis, cardiac monitoring

## Abstract

Heartbeat detection for ambulatory cardiac monitoring is more challenging as the level of noise and artefacts induced by daily-life activities are considerably higher than monitoring in a hospital setting. It is valuable to understand the relationship between the characteristics of electrocardiogram (ECG) noises and the beat detection performance in the cardiac monitoring system. For this purpose, three well-known algorithms for the beat detection process were re-implemented. The beat detection algorithms were validated using two types of ambulatory datasets, which were the ECG signal from the MIT-BIH Arrhythmia Database and the simulated noise-contaminated ECG signal with different intensities of baseline wander (BW), muscle artefact (MA) and electrode motion (EM) artefact from the MIT-BIH Noise Stress Test Database. The findings showed that signals contaminated with noise and artefacts decreased the potential of beat detection in ambulatory signal with the poorest performance noted for ECG signal affected by the EM artefacts. In conclusion, none of the algorithms was able to detect all QRS complexes without any false detection at the highest level of noise. The EM noise influenced the beat detection performance the most in comparison to the MA and BW noises that resulted in the highest number of misdetections and false detections.

## 1. Introduction

Advancement in the field of microelectronics and the computational systems has indirectly led to the evolvement of health monitoring devices for daily applications [1]. This has also enhanced the utilization of portable devices that can record ambulatory bio-signals or electrocardiogram (ECG) signals during daily-life activities such as resting, housework, exercise and other physical works. Unlike the standard ECG, the ambulatory ECG records the signal continuously over a long period out-of-hospital environment using the conventional Holter monitor [2] or the trendy wearable devices [3]. This allows the analysis of ambulatory cardiac signals that can assist in various medical applications [4,5,6,7,8] including the diagnosis of cardiac arrhythmias that can lead to sudden death or heart failure among patients [9,10].

The most important process in the monitoring system for the detection of arrhythmia is the identification of the QRS wave also recognized as the QRS complex or beat detection in ECG [11]. The beat detection is more challenging for ambulatory monitoring as the level of noise and artefacts produced during daily-life activities is greater than the monitoring process in the hospital setting. When a subject performs various high-intensity physical activities, a poor ECG signal-to-noise-ratio (SNR) may result [12]. In the ambulatory ECG, various types of noise may occur simultaneously and unpredictably that originate from stationary and non-stationary sources. Among them, baseline wander (BW), muscle artefact (MA) and electrode motion (EM) artefact which have frequency range within the frequency limit of ECG signal can manifest similar morphology as the ECG signal and can distort the clinical features of the signal which is important in recognition of various ECG arrhythmias [13,14,15].

**Figure 1:**
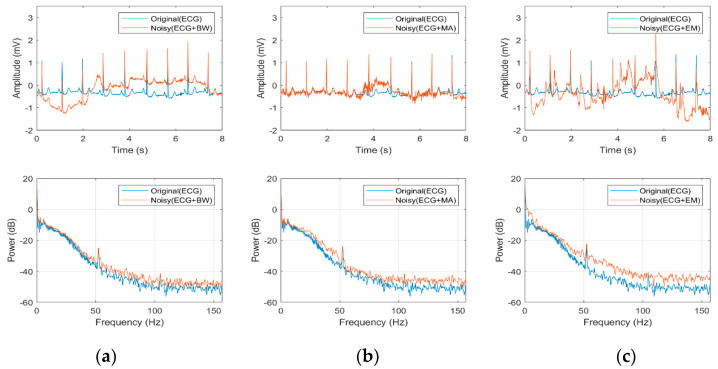
Eight-seconds of clean and noisy electrocardiogram (ECG) signals at a sampling rate of 360 Hz: (**a**) ECG with baseline wander (BW); (**b**) ECG with muscle artefact (MA); (**c**) ECG with electrode motion (EM) artefact.

The amplitude and frequency of ECG signals as affected by the artefacts in comparison to clean ECG are presented in Figure 1. The BW and abrupt drift as shown in Figure 1a could be due to the subject’s respiration movements besides being contributed by a loose or dry electrode-skin contact [16]. The BW amplitudes depend on several factors such as the subject movements, properties of electrode and skin impedance [16]. In general, the frequency of the BW is below 1 Hz but through exercise activity, the frequency of the BW in ECG recording may increase with the increasing rate of breathing. The MA or electromyogram as shown in Figure 1b was produced during a sudden body movement by the electrical activity of muscles [14]. Usually, the frequency of MA noise ranges from 20 to 1000 Hz which can cause challenges in eliminating MA without interfering with the clinical features of the ECG signal. The EM artefacts and the induced impedance change, as shown in Figure 1c, were caused by the electrode motion and have similar frequency components as the ECG signal that ranges from 1 Hz to 15 Hz [17]. Major EM artefacts can distort the signal and may lead to incorrect QRS complex and hence can cause the wrong diagnosis of arrhythmias.

Noise in ECG recordings can affect the detection process in acquiring accurate and reliable measurement of heartbeat for ECG monitoring system. Numerous studies were conducted on ECG noise analysis and different QRS detection algorithms have been developed [18,19,20,21,22]. However, most of the studies used clean data for the evaluations and assumed to reflect the overall performance of detectors. For a reliable comparison, the QRS detector performance evaluation should be carried out using the same test signal database, which was not adopted in the previous research [23]. Other studies have taken into account the influence of clinical noise with simulated noise or with experimental noise [24,25,26] which is important in an ECG signal processing task such as ECG delineation [27,28]. Nevertheless, details on the specific noise types and intensity levels that affect the QRS morphology and the beat detection performance are unavailable. Therefore, research should be conducted to evaluate the effect of noise types and intensity levels on the heartbeat detection in the ambulatory cardiac monitoring besides establishing the relationship between the beat detection and characteristics of noises especially the artefacts that can distort the ECG signal morphologically.

In this study, a methodology to compare a set of QRS detectors under different noise conditions and QRS morphologies was presented to investigate the relationship between beat detection and characteristics of noises. Experiments were performed to determine the effects of beat detector performance on clean ECG signal, heartbeat morphology, noisy signal and abnormal signal. Standard cardiac database and the simulated data using the BW, MA and EM with different levels of SNR were utilized and three algorithms were used to perform the beat detection process. The effects of noise artefacts in the ECG signals that degraded the beat detection performance were investigated.

## 2. Materials and Methods

### 2.1. Ambulatory ECG Data for Beat Detection Evaluation

Two types of ambulatory ECG signals were used in the beat detection evaluation process which were the clean ECG signal and the simulated noise-contaminated ECG signal. The clean ECG data represented the high-quality ambulatory signal and was used as a standard reference to investigate the performance of beat detection. The MIT-BIH Arrhythmia Database [29] was selected as the ECG signal was recorded in a supervised clinical environment using a Holter monitor. The database consisted of 48 recordings of ECG signals that included both normal and arrhythmic beats, each with 30-min duration with a sampling rate of 360 Hz from 47 subjects. The details of each of the 48 records are presented in Table 1. These recordings included the annotations files that contained marked locations of each QRS complex, approximately 109,505 beat annotations by two or more cardiologists.

**Figure 2:**
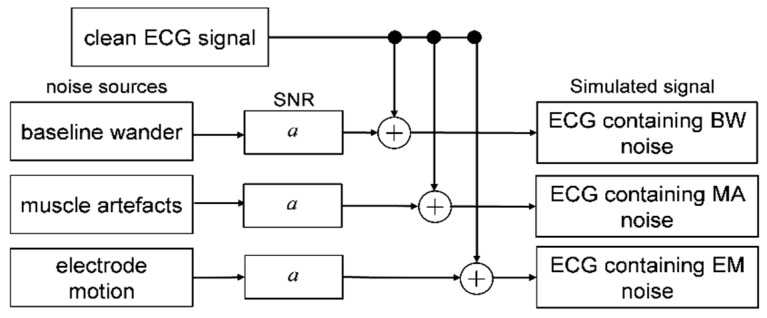
A scheme to generate the simulated ECG signal containing noise.

**Figure 3:**
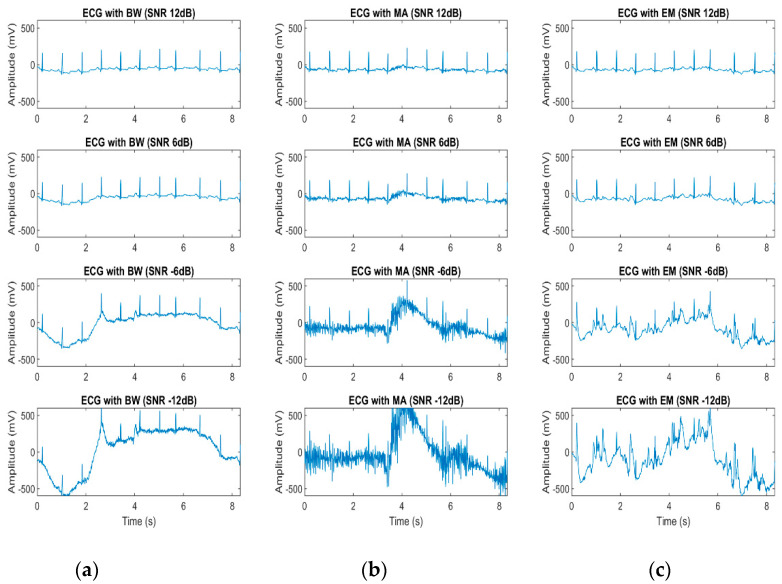
Example of simulated ECG signals that contain noise with signa-to-noise-ratio (SNR) 12, 6, −6, −12 dB: (**a**) ECG signal with BW; (**b**) ECG signal with MA; (**c**) ECG signal with EM Artefact.

The simulated noise-contaminated ECG signal was used to determine the relationship between the intensity of noises and beat detection performance using a scheme (as shown in Figure 2) where the simulated signal was produced by separately adding three sources of noise to a clean ECG signal. All 48 records from the MIT-BIH Arrhythmia Database (Table 1) [29] were used to generate the simulated noise-contaminated ECG signals. The records numbered 100 and 200 were selected for further analysis to determine the effects of beat detectors performance on noise signals and abnormal beats in ECG. The record number 100 was selected as the clean signal as it is of good quality compared to other signals and contained a few arrhythmia beats while the record number 200 was selected as an arrhythmia signal due to its dynamic signal and consisted of a fusion of arrhythmias beats (Table 1). Noise sources were added to the signal to assess the behavior of the heartbeat against the noise. The three noise sources of BW, MA, and EM from MIT-BIH Noise Stress Test Database [30] were used in this study. The noises were directly added to the aforementioned original ECGs. To simulate different levels of noise, a level of SNR from −12 to 12 dB in steps of 3 dB was used. The *SNR* was calculated using the following Equation (1),

(1) $$SNR=10\log_{10}\frac{P_{signal}}{a^{2}\times P_{noise}}$$

where 𝑃 denotes the signal power and 𝑎 refers to a scale factor. Examples of simulated ECG signals with different levels of *SNR* are shown in Figure 3.

### 2.2. Beat Detection Algorithms

Three algorithms were employed in this study to represent the beat detectors, which were the Pan Tompkins [18], the WQRS [19] and the Hamilton [20] algorithms. The main criteria for the algorithm selection were that the algorithm can be applied in a real-time system and show robust performance with the noisy and ambulatory ECG signals. The Pan Tompkins and Hamilton algorithm were implemented using the MATLAB software. The WQRS algorithm downloaded from PhysioNet website [31] is called using MATLAB scripts as the MATLAB external function. The implementation of the algorithms in this work will be made publicly available at https://github.com/Ziti481122/Effects-of-Noisy-Electrocardiogram-Signal-on-Heartbeat-Detection-Performance. The correctness of algorithm implementation was verified by analyzing the results with the same data, in this case, a record from the MIT-BIH Arrhythmia Database. It was observed that the results obtained were almost similar as reported in [18,19,20].

The Pan Tompkins algorithm [18] is one of the most well-known beat detection algorithms. This algorithm used band-pass filtering, signal differentiation, squaring, moving window integration and two sets of adaptive thresholds to filter and integrate signals for beat detection. The first step was a band-pass filtering with a passband of 5−15 Hz, which removed the BW, a 50 Hz power line interference and reduced the amplitude of T-waves. After the band-pass filtering step, the signal was then differentiated to highlight the sharp slopes of the QRS complex. To further emphasize the QRS complex, the signal was then squared to obtain positive values. The final processing step involved a moving window integration with an average window of 150 ms. This window was chosen to match the width of the widest possible QRS complex. The QRS peaks of at least 300 ms apart were identified in the pre-processed signals and classified as a noise or a QRS complex depending on the adaptive threshold.

The WQRS [19] algorithm is based on the slope and length transform of the ECG signal to identify the QRS complex. The algorithm uses low pass filters, non-linearly scaled curve length transformation and decision rules to determine the location of corresponding QRS. Instead of the band-pass filter, the low pass filter was used to eliminate the BW artefacts. The low pass filter of 16 Hz was employed to suppress the high-frequency components. Then, the ECG signal was transformed into a curve length signal using a non-linear scaling factor to enhance the QRS complex and suppress the unwanted noise. The QRS complex was determined using an adaptive threshold in the decision rules process.

The Hamilton [20] algorithm is based on the work by Pan and Tompkins [18] with alteration carried out for the pre-processing stage. The Hamilton algorithm uses band-pass filtering, differentiation, rectifying, moving window average and three rules threshold to identify the QRS complex. It differs from Pan Tompkins and WQRS algorithms where the band-pass filter of 8–16 Hz was used to remove the high- and low-frequency noises. After the band-pass filtering step, the differentiated signal was rectified instead of squaring it to highlight the QRS complex. To match the possible QRS complex in the signal, the 80 ms moving average window was used. The QRS peak of at least 300 ms away from the last detected R-peak and the peak amplitude above the detection of the adaptive threshold was classified as a QRS complex.

## 3. Results and Discussion

### 3.1. Evaluation Metrics

To validate the beat detection performance, each detected QRS peak was categorized as true positive (*TP*), false positive (*FP*) or false negative (*FN*). TP denotes the total number of QRS peaks detected as the QRS complex, FP denotes the total number of non-QRS peaks or noises detected as the QRS complex and *FN* represents the total number of QRS complexes that was not detected. Two evaluation metrics which were sensitivity (*SE*) and positive predictivity (*PP*) were calculated using Equations (2) and (3), respectively [11]. The SE denotes the percentage of true beats that are correctly detected by the algorithm, whereas the PP denotes the percentage of detected true beats. These two metrics were calculated using the total number of *TP*, *FN* and *FP*.

(2) $$SE=\frac{TP}{TP+FN}\times 100\%$$

(3) $$PP=\frac{TP}{TP+FP}\times 100\%$$

### 3.2. Effect of Beat Detector Performance on a Clean ECG

**Figure 4:**
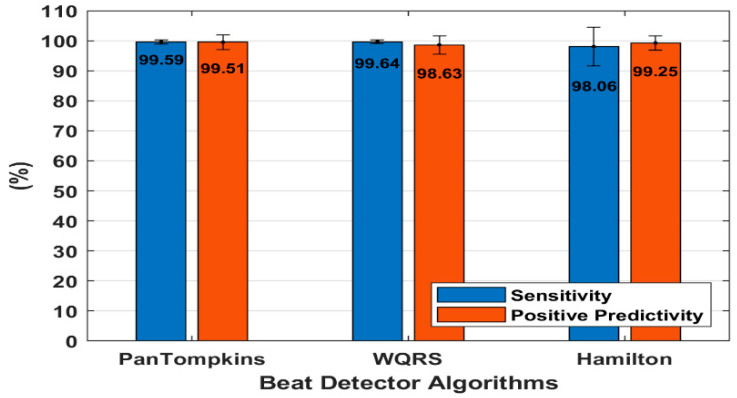
Beat detector algorithm performance on a clean data.

The heartbeat detector performance on a clean ambulatory ECG signal was evaluated using 48 records from the MIT-BIH Arrhythmia Database (Table 1) [29]. Figure 4 presents the average performance of the three algorithms of beat detectors on all 48 ECG records. There was no significant difference found in the performances of these algorithms when using a clean ECG. All the algorithms produced SE and PP with an average above 98% which indicated good performance of the algorithms for both clean and diverse clinical ECG signals from 47 subjects.

It was observed that the Hamilton algorithm has a good *PP*, however, the *SE* decreased which indicated the algorithm’s sensitiveness towards abnormalities of heart rhythm. Although the WQRS algorithm was capable to detect the correct QRS peak with the highest total SE of 99.64%, the algorithm was also sensitive to noise. The WQRS algorithm often detected false peak as the QRS complex thus producing a low *PP* (Figure 4). It was also found that the Pan Tompkins algorithm has the stability to perform beat detection compared to the other two methods with 99.59% of SE and 99.51% of *PP*, respectively.

All beat detector algorithms performed well for most of the records in the MIT-BIH Arrhythmia Database [29]. Nevertheless, in this database, there were a few records, such as record numbers 105, 108, 121, 200, 202, 207, and 217, that have dynamic signals due to abnormal beats and noise effects. Previous research also used these records to assess the noise robustness [32,33]. According to the PhysioNet web-based resource [34], the signal from record 207 is the extremely difficult record in the MIT-BIH Arrhythmia Database due to the predominant rhythm of abnormal beats in the signal. In this study, comparison of the algorithm performance for the few difficult records such as record numbers 105, 108, 121, 200, 202, 207, and 217 was also carried out as shown in Table 2.

The findings showed that the beat detector can handle both normal and abnormal beat signals such as record numbers 200, 202, 207, and 217. The signal from the record 200 indicated a normal and combination of ventricular beats, while the signal from the record 202 showed a normal, atrial premature and premature ventricular contraction beat. The ECG signal of the record 217 composed of normal beats with a fusion of paced and premature ventricular contraction beats. Results showed that the beat detectors performance with this signal resulted in the percentage of *SE* and *PP* above 98.98% and 98.2%, respectively. It was also observed that the signal from the record 121 was distorted by the BW, however, this did not affect the detection performance. However, the performance of the beat detector degraded especially with the signal from the record 108 that was despoiled by MA and low amplitude, and the signal from the record 105 that was contaminated with high-grade noise.

### 3.3. Effect of Noisy Signal on Heart Beat Morphology

**Figure 5:**
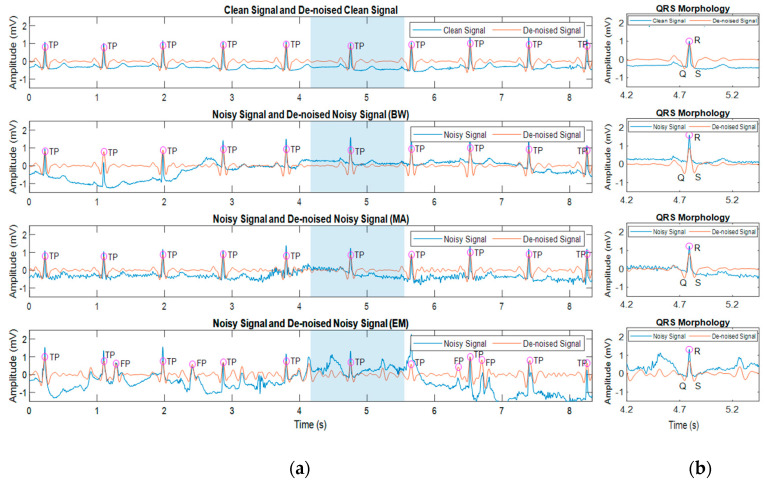
Visual evaluation of the ECG morphologies as affected by clean and noisy signals with a SNR of 0 dB (**a**) ECG Signal and De-Noised ECG Signal; (**b**) the QRS morphologies of heartbeat.

Effects of noise towards heartbeat morphology in a noisy signal were also studied. Simulated signals using the record number 100 from MIT-BIH Arrhythmia Database that were contaminated with BW, MA, and EM with SNR 0 dB were evaluated separately to investigate the heartbeat morphologies as affected by noisy signals. The Pan Tompkins [18] algorithm was chosen due to the comprehensive approach to reduce the interferences and to avoid false detection of QRS complexes in ECG signals. The algorithm also has higher accuracy for various beat morphologies than the other traditional real-time methods [35]. The QRS characteristics of heartbeat morphologies were evaluated after the band-pass filtering process with 5 to 15 Hz and adaptive thresholds of Pan Tompkins algorithm to reduce the destruction caused by the noises and identify the true beats in ECG signals. Figure 5 shows the ECG morphologies as affected by noisy and de-noised signals.

As can be seen in Figure 5, the blue, and orange signal represents the signal before and after the filtering process, respectively. The TP denotes true positive while FP denotes the false detection. The blue areas represent the QRS morphology in Figure 5b. The findings showed that the ECG morphologies were distorted by the different noises. The BW noise due to the subject’s respiration movements presented an abrupt drift in the signal that introduced some interference to the signal. The MA noise with the high-frequency range interfered the morphological features in the signal. Besides, the ECG information changed when motion artefacts were introduced to the signal which caused irregularities in the ECG morphology. The difference in frequency ranges of BW, MA and EM artefact led to distorted ECG signal morphologies.

In this study, the filtering process smoothened the ECG morphology and enhanced the QRS complex. Although the signal contaminated with BW and MA degraded the morphology, the algorithm managed to discover the QRS complex after the filtering process. However, the irregularities caused by the EM artefact cannot be solved using the band-pass filter, thus resulting in a false detection as shown in Figure 5. It can be observed that the ECG signals contaminated with EM noise have the poorest signal compared to the other noises. The presence of undesired interferences from high-frequency noises caused a serious problem in the ECG diagnosis [5].

### 3.4. Effect of Beat Detector Performance on the Noisy Signal of Record 100

**Figure 6:**
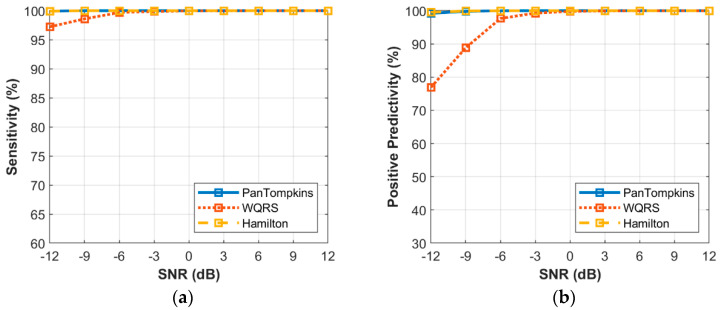
Relationship between the performance of beat detection and BW for the record 100: (**a**) sensitivity with SNR; (**b**) positive predictivity with SNR.

**Figure 7:**
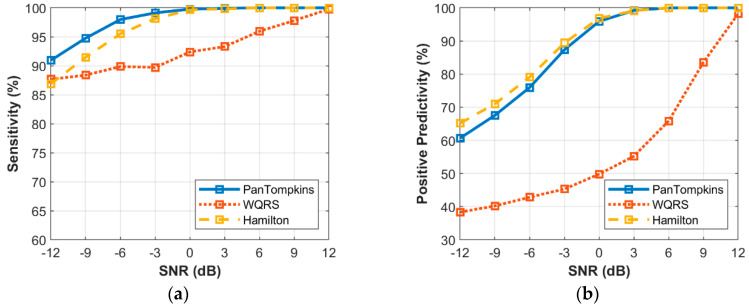
Relationship between the performance of beat detection and MA for the record 100: (**a**) sensitivity with SNR; (**b**) positive predictivity with SNR.

**Figure 8:**
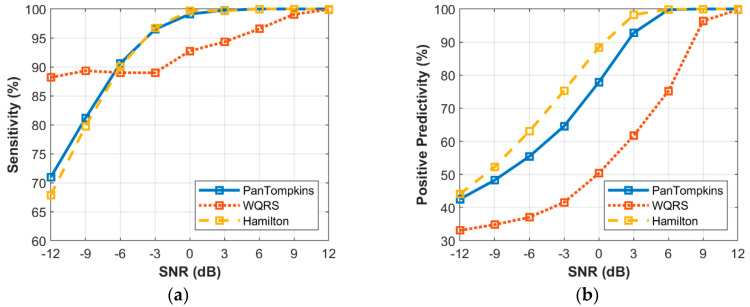
Relationship between the performance of beat detection and EM artefact for the record 100: (**a**) sensitivity with SNR; (**b**) positive predictivity with SNR.

Effect of a heartbeat detector on the different intensity of noise was identified. The simulated signals contaminated with BW, MA and EM were used to investigate the relationship between the beat detection performance and the intensity of noise as exhibited in Figure 6, Figure 7 and Figure 8. To evaluate the effects of noisy signal, the record number 100 from MIT-BIH Arrhythmia Database was used.

The relationship between the different intensity of BW noise and the performance of beat detection on the signal is shown in Figure 6. In response to the sensitivity of the three algorithms, at SNR levels above −6 dB, all the algorithms scored very well. At levels below a SNR of −6 dB, the beat detector performance decreased, especially for the WQRS algorithm where the SE was lower, 97.23% at a SNR of −12 dB which indicated the algorithm was sensitive to BW noise. In contrast, the Pan Tompkins and Hamilton algorithms possessed SE lower than a SNR of −9 dB, where the SE of both the Pan Tompkins and Hamilton algorithms decreased to 99.92% and 99.91%, respectively. In terms of PP, the Hamilton and Pan Tompkins algorithms have a significantly better performance with 99.52% and 99.21%, respectively in −12 dB SNR of BW noise compared to the WQRS algorithm that has low performance with 76.9%.

Figure 7 shows the relationship between the different intensity of MA noise and the performance of beat detection on the signal. It was found that below SNR of 3 dB, the performance of beat detector continued to decrease with the drop in SNR value with SE of 90.94% and 86.89% as produced by the Pan Tompkins and Hamilton algorithms, respectively at a SNR of −12 dB. The WQRS algorithm showed that the detector was very sensitive and unstable with MA and resulted in lower SE and PP performance. As for the PP, MA affected the performance of the Pan Tomkins and Hamilton algorithms with a SNR value below 3 dB. However, the Hamilton algorithm has a better PP (65.16%) at a SNR of −12 dB compared to the other two algorithms.

The relationship between the intensity level of EM noise and the performance of beat detection is shown in Figure 8. The signal that was contaminated with the EM artefact below a SNR of 0 dB degraded the detection performance of the Pan Tompkins and Hamilton algorithms. At a SNR of −12 dB, the Pan Tompkins and Hamilton algorithms decreased the SE to 70.96% and 67.88%, respectively, lower than the SE of WQRS algorithm which was 88.17%. This could be attributed to high false-positive detection in the signal with high-frequency noises from EM artefact (Figure 8b) which decreased the PP of the detector performance. All three algorithms, the Hamilton, the Pan Tompkins and the WQRS produced low PP with 44.05%, 42.54% and 33.09%, respectively at a SNR of −12 dB.

### 3.5. Effect of Beat Detector Performance on Noisy Abnormal Signal of Record 200

**Figure 9:**
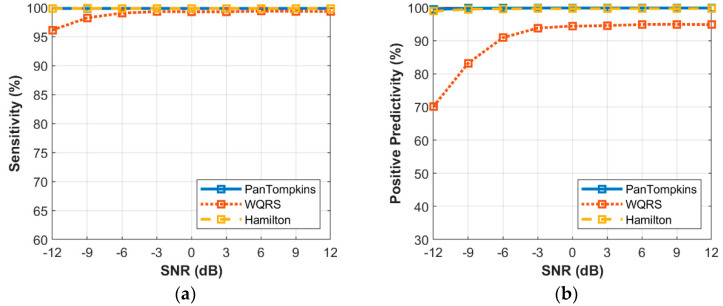
Relationship between the performance of beat detection and BW with abnormal signal for the record 200: (**a**) sensitivity with SNR; (**b**) positive predictivity with SNR.

**Figure 10:**
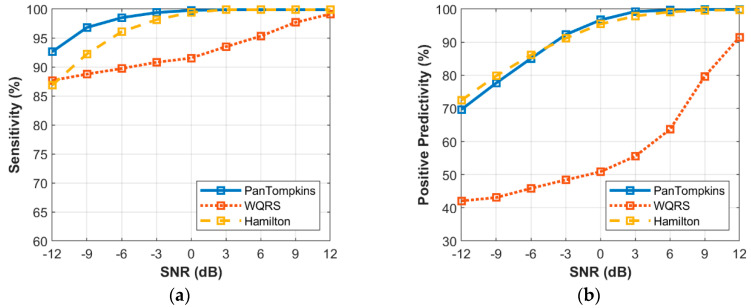
Relationship between the performance of beat detection and MA with abnormal signal for the record 200: (**a**) sensitivity with SNR; (**b**) positive predictivity with SNR.

**Figure 11:**
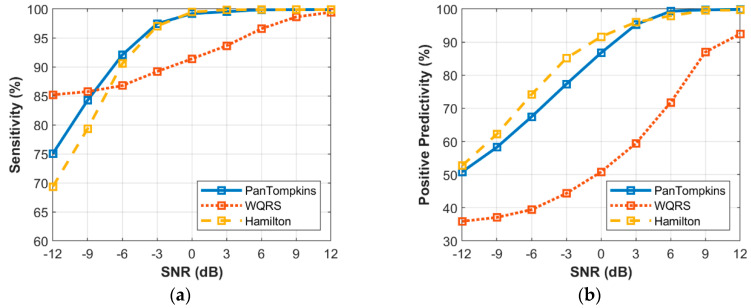
Relationship between the performance of beat detection and EM artefact with abnormal signal for the record 200: (**a**) sensitivity with SNR; (**b**) positive predictivity with SNR.

The effect of a heartbeat detector on different levels of noise in the ECG signal that consisted of both abnormal or arrhythmia beats was determined. The simulated signals that contaminated with BW, MA and EM were used to evaluate the effects of detection on the intensity of the noise signal. Figure 9, Figure 10 and Figure 11 demonstrate the relationship between the performance of beat detection and the level of noise. To evaluate the effects of noise contamination on arrhythmia signal, the record number 200 from MIT-BIH Arrhythmia Database was used. As listed in [32], this record has a dynamic signal and consists of a fusion of arrhythmias and normal beats.

It can be observed that the noise in the abnormal signal destructed the heartbeat rhythm of arrhythmias morphology thus degrading the ECG signal quality and affected the beat detection performance (Figure 9). The BW noise affected the beat detection process of the Pan Tompkins and the Hamilton algorithms less compared to the WQRS algorithm. The *SE* as influenced by the Pan Tompkins, the Hamilton and the WQRS algorithms were 99.85%, 99.81% and 96.12%, respectively, with PP of 99.39%, 98.90% and 70.11%, respectively, at a *SNR* of −12 dB.

Below a SNR of 3 dB, the detection of signal contaminated with MA noises in abnormal signal reduced the SE (Figure 10a). At a SNR of −12 dB the Pan Tompkins, the WQRS and the Hamilton algorithms resulted in a SE of 92.62%, 87.66% and 86.85%, respectively, while at a SNR of −12 dB, the PP decreased to 69.66%, 42.07% and 72.45%, respectively (Figure 10b). However, the signal contaminated with EM noises affected heartbeat detection. As shown in Figure 11, the EM artefact produced a lower performance of heartbeat detection with a lower SE of 69.36% at a SNR of 12 dB using the Hamilton algorithm. In contrast, the lower PP at a SNR of −12dB for a signal contaminated with EM artefact was 35.9% using the WQRS algorithm that indicated the inability of this algorithm to manage the false positive in the detection process and performance maintenance.

### 3.6. Effect of Beat Detector Performance on Noisy Signal of all Records from the MIT-BIH Database

**Figure 12:**
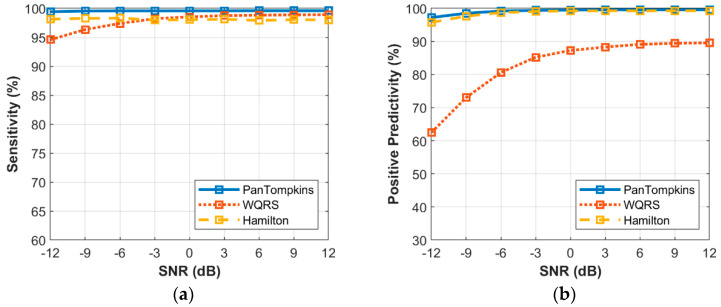
Relationship between the performance of beat detection and BW for all the 48 records: (**a**) sensitivity with SNR; (**b**) positive predictivity with SNR.

**Figure 13:**
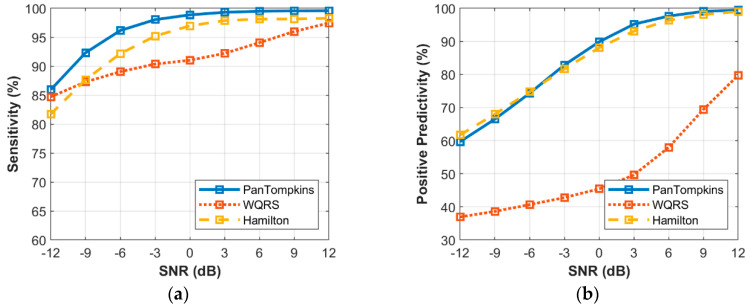
Relationship between the performance of beat detection and MA for all the 48 records: (**a**) sensitivity with SNR; (**b**) positive predictivity with SNR.

**Figure 14:**
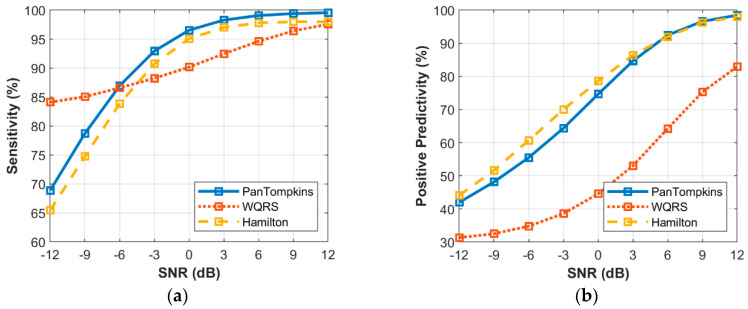
Relationship between the performance of beat detection and EM artefact for all the 48 records: (**a**) sensitivity with SNR; (**b**) positive predictivity with SNR.

The effect of the different intensity of noise on the heartbeat detector method in 48 records from the MIT-BIH Arrhythmia Database was identified. The simulated signals that were contaminated with BW, MA and EM were investigated. The relationships between the beat detection performance and the intensity of noise are exhibited in Figure 12, Figure 13 and Figure 14. The SE and PP in Figure 12, Figure 13 and Figure 14a,b represent the average performance of beat detection on all 48 records of noisy ECG signals.

Figure 12 shows the relationship between the different intensity of BW noise and the average performance of beat detection. In response to the average sensitivity of the three algorithms, at the highest level of noise, all the algorithms were not able to detect all QRS complexes. The WQRS algorithm resulted in low *SE* (94.58%) at *SNR* of −12 dB while the Pan Tompkins and Hamilton algorithms yielded a better performance with 99.42% and 98.13%, respectively. In terms of average PP, the Pan Tompkins and Hamilton algorithms had a significantly better performance with 97.21% and 95.74%, respectively, at −12 dB SNR of BW noise compared to the WQRS algorithm (62.45%).

The relationship between the intensity level of MA noise and the average performance of beat detection is shown in Figure 13. The MA noise affected the *SE* and *PP* of the beat detection process of all algorithms. At a *SNR* of −12 dB, the average *SE* of the Pan Tompkins, Hamilton and WQRS algorithms were 85.94%, 81.74% and 84.71%, respectively and that of the PP was 59.68%, 61.74% and 36.95%, respectively. The WQRS algorithm showed that the detectors produced high false-negative detections and resulted in lower average *PP* performance at all levels of SNR compared to the Pan Tompkins and Hamilton algorithms (Figure 13b).

Figure 14 shows the signals contaminated with EM artefacts have degraded the detection performance of all algorithms. At a SNR of −12 dB, the Pan Tompkins and Hamilton algorithms decreased the SE to 68.85% and 65.44%, respectively, which were lower than the corresponding value of the WQRS algorithm (84.10%). As for the *PP*, the EM affected the average performance of algorithms at all *SNR* values. All algorithms, Hamilton, Pan Tompkins and WQRS, produced low *PP* with 44.05%, 42.54% and 33.09%, respectively, at SNR of −12 dB.

As shown by Figure 12, Figure 13 and Figure 14, signals contaminated with BW, MA and EM artefacts degraded the detection performance of the Pan Tompkins, Hamilton and WQRS algorithms. The analysis on the 48 records from the MIT-BIH Arrhythmia Databases showed that the noisy signal decreased the beat detection performance, with low average *SE* and *PP* at the lowest *SNR*, compared to the average detection in clean ECG signals (Figure 4). The average detection performance showed the highest influence by MA and EM artefacts, with the sensitive WQRS algorithm being most affected by the noisy signal.

## 4. Conclusions

With ambulatory cardiac monitoring systems becoming more widespread, the detection performance of heartbeat as a dominant feature in classifying cardiac disease especially arrhythmia, cannot be ensured and is still questionable in a noisy signal. To overcome the issues, robust heartbeat algorithms are required for the signal generated in the ambulatory environment. The confirmation about which type of noise that could distort the ECG signal, and the algorithm performance must be clarified.

In this study, the relationship between the ECG noise and heartbeat detection for ambulatory cardiac monitoring was investigated using heartbeat detection algorithms for both clean and noise-simulated ECG signals that were contaminated with BW, MA and EM artefacts. There was no significant difference found in the performance of beat detection algorithms when the clean signal was used. The beat detector was able to handle the high-quality signals from the MIT-BIH Arrhythmia database that has dynamic signal due to abnormal beats, noise and artefacts effects.

The experimental results on noisy signal showed valid beat detection performance. The findings implied that signal contaminated with noise and artefacts degraded the ECG morphology and decreased the potential of beat detection in the ambulatory signal. This is represented by the relationship between the noise types and the level of *SNR* intensity, and confirmed by the performance of the average *SE* and *PP* of the algorithms used in the experiments. Based on the results, none of the algorithms were able to detect all the QRS complexes without any false positive and false negative at the highest level of noise indicating the weakness of the Pan Tompkins, the WQRS and the Hamilton algorithms. The Pan Tompkins algorithm showed the best performance of detection when dealing with noisy signals, followed by the Hamilton algorithm, while the WQRS algorithm showed the poorest performance.

The relationship study between the characteristics of ECG noises and the beat detection indicated that the BW has a lesser influence on the beat detection performance except with the more sensitive WQRS algorithm. Meanwhile, the EM artefacts have the highest influence on the detection algorithm, followed by MA and BW. Higher interferences that degraded the detection performance were mainly due to MA and EM artefacts. The higher intensity of MA and EM artefacts contributed to the false positive and false negative values that affected the percentage of QRS complexes detected. However, the EM artefacts contributed to the poorest detection performance which was proved by the lower performance of *SE* and *PP* in the high noise signal and the distorted ECG morphology. This has led to the highest number of misdetections and false detections. Further improvements should consider effect of MA and EM artefacts in ECG signals to deal with the false detection of the QRS complex in order to improve the detection performance.

Future work will focus on applying motion artefact reduction algorithms to overcome the effect of MA and EM artefacts in the detection of heartbeats. This will lead to developing a robust heartbeat algorithm in the cardiac ambulatory monitoring system.

## Figures and Tables

**Table 1:** The MIT-BIH Arrhythmia Database.

Record	Beats						Record	Beats					
	Total	N ^1^	S ^2^	V ^3^	F ^4^	Q ^5^		Total	N ^1^	S ^2^	V ^3^	F ^4^	Q ^5^
100	2273	2239	33	1	0	0	201	1963	1635	128	198	2	0
101	1865	1860	3	0	0	2	202	2136	2061	55	19	1	0
102	2187	99	0	4	56	2028	203	2980	2529	2	444	1	4
103	2084	2082	2	0	0	0	205	2656	2571	3	71	11	0
104	2229	163	0	2	666	1398	207	1860	1543	107	210	0	0
105	2572	2526	0	41	0	5	208	2955	1586	2	992	373	2
106	2027	1507	0	520	0	0	209	3005	2621	383	1	0	0
107	2137	0	0	59	0	2078	210	2650	2423	22	195	10	0
108	1774	1740	4	17	2	0	212	2748	923	1825	0	0	0
109	2532	2492	0	38	2	0	213	3251	2641	28	220	362	0
111	2124	2123	0	1	0	0	214	2262	2003	0	256	1	2
112	2539	2537	2	0	0	0	215	3363	3195	3	164	1	0
113	1795	1789	6	0	0	0	217	2208	244	0	162	260	1542
114	1879	1820	12	43	4	0	219	2154	2082	7	64	1	0
115	1953	1953	0	0	0	0	220	2048	1954	94	0	0	0
116	2412	2302	1	109	0	0	221	2427	2031	0	396	0	0
117	1535	1534	1	0	0	0	222	2483	2274	209	0	0	0
118	2278	2166	96	16	0	0	223	2605	2045	73	473	14	0
119	1987	1543	0	444	0	0	228	2053	1688	3	362	0	0
121	1863	1861	1	1	0	0	230	2256	2255	1	0	0	0
122	2476	2476	0	0	0	0	231	1571	1568	1	2	0	0
123	1518	1515	0	3	0	0	232	1780	398	1382	0	0	0
124	1619	1536	31	47	5	0	233	3079	2230	7	831	11	0
200	2601	1743	30	826	2	0	234	2753	2700	50	3	0	0

^1^ Normal (N), ^2^ Supraventricular Ectopic (S), ^3^ Ventricular Ectopic (V), ^4^ Fusion (F), ^5^ Unknown (Q).

**Table 2:** Comparison of the beat detector performances for ECG records 105, 108, 121, 200, 202, 207 and 217.

Record	Pan Tompkins [18]		WQRS [19]		Hamilton [20]	
	*SE* (%)	*PP* (%)	*SE* (%)	*PP* (%)	*SE* (%)	*PP* (%)
105	99.46	98.27	98.83	92.10	99.57	98.88
108	99.77	83.27 ^1^	99.38	84.19 ^1^	99.32	99.38
121	99.89	100	99.79	99.73	99.95	100
200	99.85	99.85	99.85	99.31	99.85	99.73
202	99.53	100	99.81	99.95	99.67	100
207	98.98	99.68	99.41	98.40	99.25	99.84
217	99.82	99.91	99.55	98.30	99.18	99.64

^1^ Low positive predictivity.
